# Differences in the Prolonged Effectiveness Between Two Denture Adhesives With Different Rheological Characteristics

**DOI:** 10.7759/cureus.79248

**Published:** 2025-02-18

**Authors:** Tetsurou Torisu, Junko Sato, Kazumi Yamaguchi, Tomoyasu Mori, Hitomi Okazaki, Kazuhiro Yoshida, Hiroshi Murata

**Affiliations:** 1 Department of Prosthetic Dentistry, Nagasaki University Graduate School of Biomedical Sciences, Nagasaki, JPN; 2 Oral Care Research and Development Group, Kobayashi Pharmaceutical Co. Ltd., Tokyo, JPN

**Keywords:** adhesive strength, denture adhesive, denture movement, long-term effectiveness, material characteristics

## Abstract

Aim

This study aimed to investigate the differences in long-term effectiveness between two marketed denture adhesives: adhesive A (tough-grip cream; Kobayashi Pharmaceutical Co., Ltd., Osaka, Japan) and adhesive B (tough-grip cream A; Kobayashi Pharmaceutical Co., Ltd.) with different material science characteristics.

Materials and methods

This study consists of the following two parts: Experiment 1 - measuring material characteristics in vitro; Experiment 2 - measuring the denture movement and subjective evaluation in participants. The adhesion strength, viscosity, dynamic viscoelastic properties, and temporal changes in adhesion strength were measured in vitro. The study also investigated denture movement and subjective evaluations (stableness of the denture, duration of stableness, inhibitory effect on wobble, and inhibitory effect on slippage) after participants used the adhesives for a prolonged period (seven hours). Twelve participants were randomly divided and assigned into two protocols: protocol A (adhesive A followed by adhesive B) and protocol B (adhesive B followed by adhesive A).

Results

In the in vitro study, no significant differences were observed in adhesion strength or viscosity between the two adhesives. However, the storage moduli, loss moduli, and loss tangents exhibited significant differences. With respect to changes in temporal adhesion strength, adhesive B exhibited significantly higher values in the later cycles than did adhesive A. In the in vivo study, adhesive B tended to be preferred only in terms of the “duration of stableness” on the questionnaire, more than adhesive A, when the participants compared the two adhesives after they completed the two protocols. No statistical significance was observed between the two adhesives in vertical denture movement or subjective evaluations by visual analog scale (VAS). Vertical movement significantly decreased, and subjective denture stability significantly increased after five minutes compared to baseline for both adhesives. These effects were maintained seven hours later.

Conclusion

Our results indicate that both adhesives have sufficient long-lasting effects in clinical conditions for patients under conditions similar to those in this study, despite demonstrating different material characteristics in some factors in the in vitro study. Denture adhesives contribute to an improved quality of life and self-esteem, even for well-fitting dentures. Further studies are required to clarify the differences between denture adhesives under more severe oral conditions.

## Introduction

The global incidence of edentulism has declined; however, many countries still have large numbers of edentulous individuals [1-3]. Dental implants have markedly improved therapeutic effects; however, many edentulous patients cannot receive implants due to several factors, such as their general physical condition, the quality of denture-supporting tissues, inadequate dental care facilities, or financial reasons. For these patients, conventional complete dentures remain an important option [1].

Several studies have examined the effectiveness of denture adhesives [2-12]. These studies reported that denture adhesives improve retention, stability [11], masticatory performance [6,10], accumulation of food particles under dentures [11], self-confidence [7], satisfaction with dentures, and oral health-related quality of life (OHRQoL) [9,12]. With an increased focus on self-esteem and social interaction to improve quality of life, the demand for complete dentures has risen. Consequently, we may observe an increased use of denture adhesives among the “young-elderly” denture-wearing population [3]. In this context, it is important to choose denture adhesives that provide long-term stability.

To the best of our knowledge, no study has investigated the change in effectiveness when denture adhesives are used for a long time. Moreover, no study has compared changes in the effectiveness of denture adhesives with different material science characteristics.

Recently, an improved cream-type product has been developed with the aim of achieving a long-lasting effect. Therefore, this study aimed to investigate the differences in material characteristics between two denture adhesives (the existing and improved products) in vitro. Additionally, we investigated denture movement and subjective evaluations after participants used the adhesives for a prolonged period (seven hours). We hypothesized that the improved product would show better, longer-lasting effects both in vitro and in vivo.

## Materials and methods

This study consists of the following two parts: Experiment 1 - measuring material characteristics in vitro; Experiment 2 - measuring denture movement and subjective evaluation in participants.

Two marketed denture adhesives, tough-grip cream (denture adhesive A: existing product; Kobayashi Pharmaceutical Co., Ltd., Osaka, Japan) and tough-grip cream A (denture adhesive B: improved product; Kobayashi Pharmaceutical Co., Ltd.), were used in both experiments. The improved product (tough-grip cream A) aims to provide long-lasting effects. Both denture adhesives are composed of the base (white petrolatum and soft liquid paraffin), adhesive (carboxymethyl cellulose sodium, or CMC-Na), and other components. The composition ratios of each product were not made public. It is reported that the adhesion strength increased with an increasing percentage of CMC-Na [13]; therefore, it is assumed that the blending ratio of CMC-Na was changed to be higher in this improved product.

Experiment 1 (measuring material characteristics in vitro)

Adhesion Strength

The adhesion strengths of the two denture adhesives were measured using a universal tester (EZ Test/CE; Shimadzu Corporation, Kyoto, Japan) according to International Organization for Standardization (ISO) specification 10873 [14]. Briefly, the sample holder, made of acrylic resin, had a hole with a diameter of 22 ± 1 mm and a depth of 0.5 ± 0.1 mm. One of the denture adhesives was placed in the hole. A constant load of up to 10.0 ± 0.2 N was applied by the pressure-sensitive shaft with a circular base and a diameter of 20 ± 0.5 mm. The load was held for 30 seconds to allow for stress relaxation. The shaft was then pulled in the opposite direction at a crosshead speed of 5 mm/min, and the maximum adhesion strength value was recorded. Measurements were performed five times for each adhesive at 37 ± 2°C.

Viscosity and Dynamic Viscoelastic Characteristics

Viscosity and dynamic viscoelastic characteristics were measured using a controlled stress rheometer (Discovery HR-2; TA Instruments, New Castle, DE, USA) according to a previous study [13,15]. A round, parallel plate with a diameter of 20 mm was used. The gap between the round plate and the top plate of the body was set to 1.0 mm. For viscosity, measurements were performed at a shear rate ranging from 0.1 to 100 sec⁻¹ at 37°C, with values at 1 sec⁻¹ used for subsequent analyses. For viscoelastic characteristics, measurements were performed at oscillation frequencies ranging from 0.01 to 100 Hz at 37°C, with values at 1 Hz used for subsequent analyses. The parameters measured included storage modulus (G’), loss modulus (G”), and loss tangent (tan delta = G”/G’).

Temporal Change in Adhesion Strength

**Figure 1:**
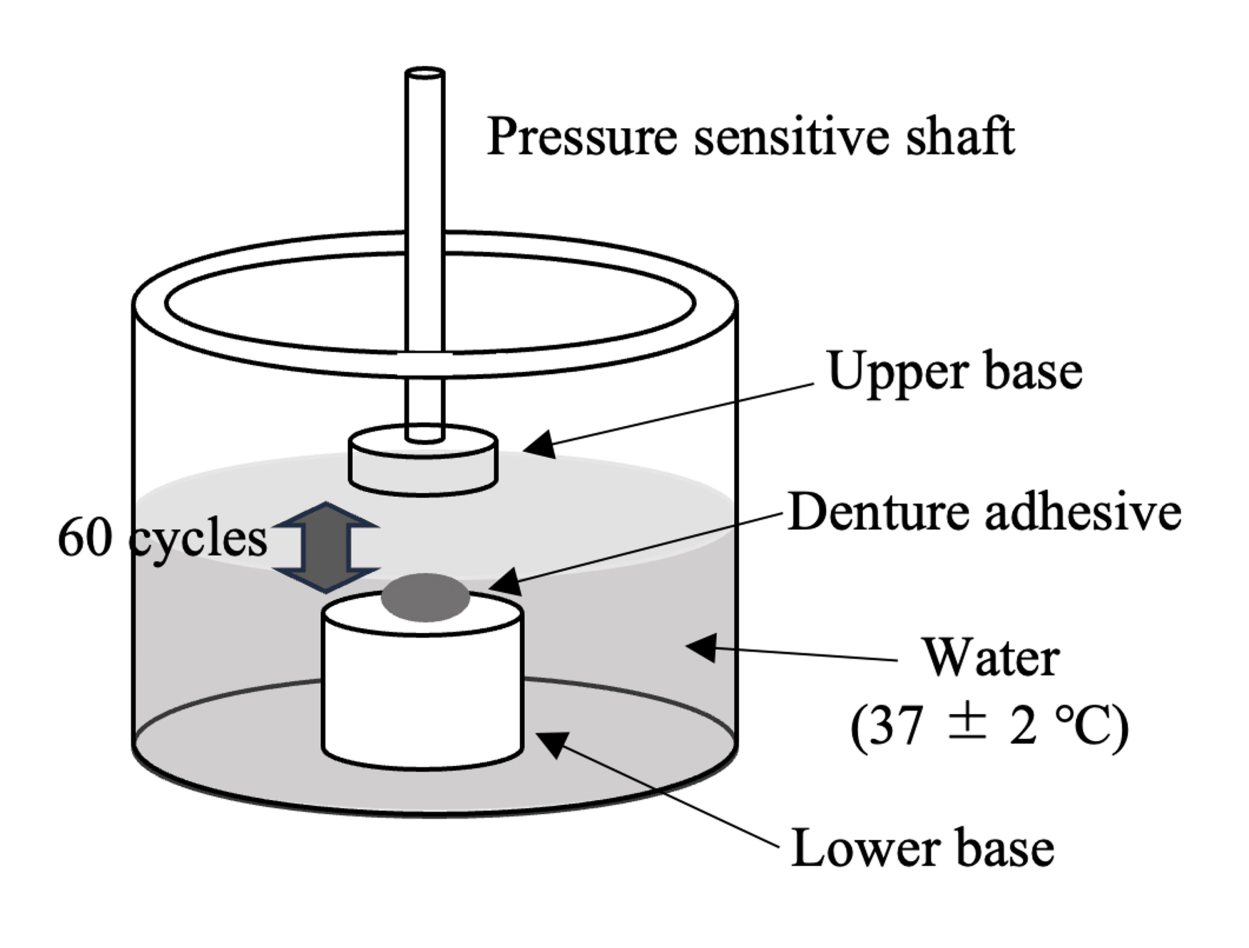
Experimental setting for measurement of adhesive strength Image credit: This image has been created by the authors of this article.

Temporal changes in the adhesion strength against repeated tensile tests were measured using a rheometer (CR-500DX-L; Sun Scientific Co. Ltd., Tokyo, Japan) according to the specification of the Japanese Industrial Standards (JIS) T 6525-1. A pressure-sensitive shaft with a circular upper base, 20.0 ± 0.5 mm in diameter and made of acrylic resin, was fixed to the load detector. One of the denture adhesives (0.1 g) was placed on the lower base, also made of acrylic resin, and water was poured around the base. The measurements were performed in water. The temperature of the water was kept at 37 ± 2°C. A constant load of up to 9.8 ± 0.2 N was applied by the pressure-sensitive shaft prior to tensile measurement. The tensile test was performed at a crosshead speed of 50 mm/min. The maximum tensile force was recorded 60 times for each trial. Each trial lasted approximately seven minutes. The distance moved by the shaft was approximately 3 mm. Ten trials were conducted for each adhesive. For each trial, the denture adhesives between the bases were replaced with a new sample (Figure 1).

Experiment 2 (measuring the denture movement and subjective evaluations in participants)

Participants

Twelve patients with a maxillary complete denture, five males and seven females (mean age: 72.3 ± 10.9; range: 45-83), participated in this study. The patients visited Nagasaki University Hospital, Nagasaki, Japan, for denture recall. The mean duration of use for maxillary complete dentures was 2.4 years (±3.3; range: 1.5 months to 10 years). The inclusion criteria included adult patients (≥20 years) who were using maxillary complete dentures. The patients who were in the maintenance period after adjustment by specialists participated in this study.

The exclusion criteria were as follows: (a) no use of dentures during meals; (b) pain or discomfort in the orofacial area; (c) dysfunction in the masticatory system; (d) constant use of denture adhesives; (e) use of tooth attachment or implants for maxillary complete dentures; (f) serious systemic illness; (g) difficulty responding to the questionnaire; and (h) allergy to flour and/or gelatin, as we used gummy jelly containing them as the test food.

Regarding the mandibular conditions of the participants, seven patients used removable partial dentures; four patients used complete mandibular dentures; and one patient had only a bridge-type prosthesis without the use of removable dentures.

This study was approved by the Nagasaki University Hospital Clinical Research Ethics Committee (Approval No. 22041802). This study was registered with the University Hospital Medical Information Network (UMIN000047108). All participants provided written informed consent in accordance with the Declaration of Helsinki and were informed that they were free to withdraw from the experiment at any time. Data collection was done during the approved period (from June 30, 2022, to December 16, 2022).

Study Design

**Figure 2:**
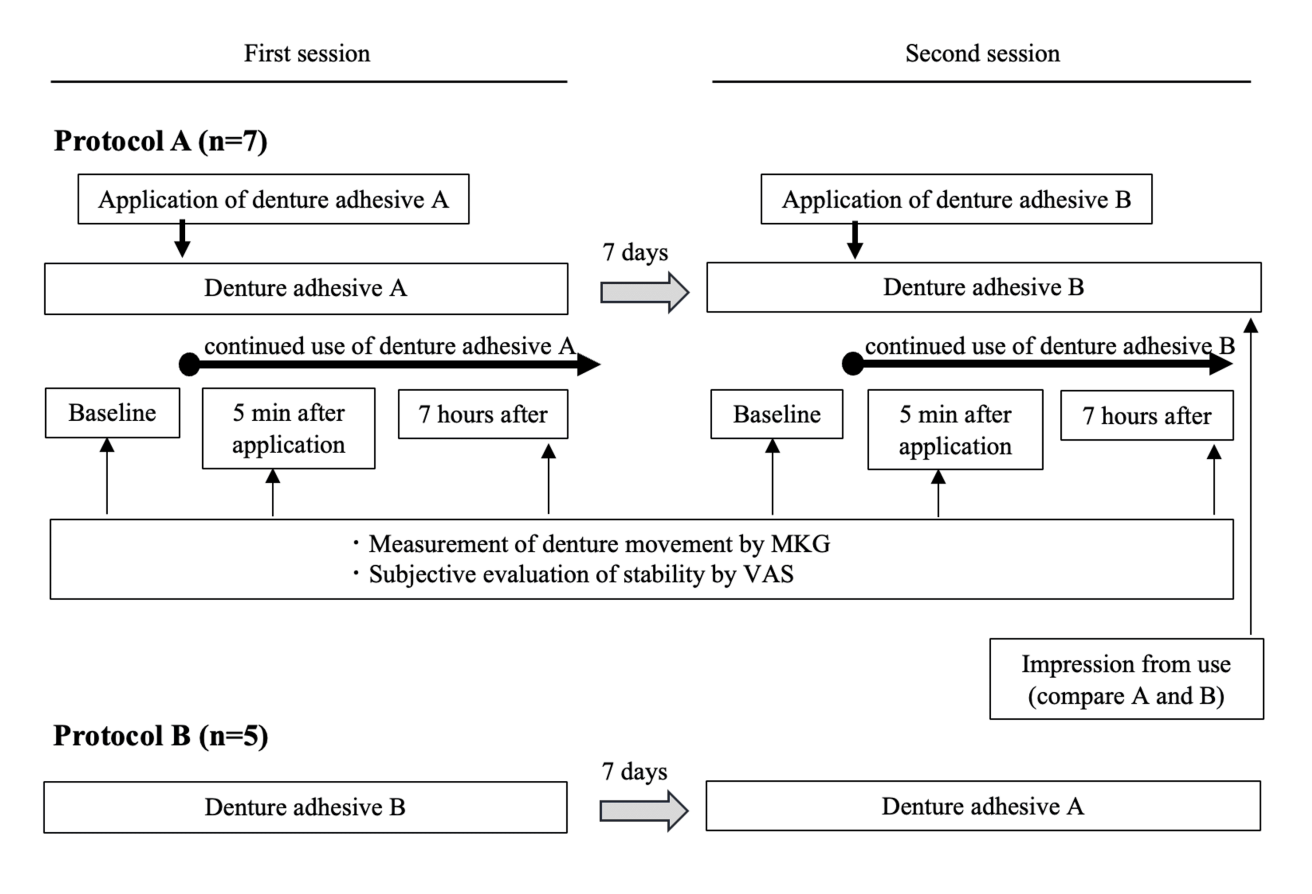
Study design of the Experiment 2 MKG, Mandibular Kinesiograph; VAS, Visual Analog Scale

Experiment 2 was designed as a crossover trial consisting of two sequences (protocols A and B). In protocol A, denture adhesive A (tough-grip cream) was used in the first session, followed by denture adhesive B (tough-grip cream A) in the second session. The interval between the sessions was seven days. Protocol B was performed in the reverse order. Seven participants were allocated to protocol A and five to protocol B, totaling 12 participants (Figure 2).

The participants visited the hospital in the morning. At the beginning of the first session, the habitual masticatory side was identified. The participants were asked to chew a dental cotton roll freely for 10 seconds, repeating this process three times. The side where the cotton roll was applied more than twice was designated as their habitual masticatory side.

Commercially available gummy jelly (fruit juice gummy; Meiji Co., Ltd., Tokyo, Japan) was used as the test food. The participants chewed one gummy jelly freely on the habitual masticatory side using the jaw-tracking system described below. Before the baseline measurements, the participants were asked to chew one or two gummy jellies to familiarize themselves with the experimental conditions. Baseline data were then collected before the application of the denture adhesive. An electronic weighing instrument was used to measure 0.5 g of the adhesive. After baseline measurements, 0.5 g of one of the two adhesives was applied to the maxillary denture at three locations: the midline of the anterior tooth and the molar areas on both sides. After applying the adhesive, the participants were instructed to keep their mouths closed with light clenching. A second measurement was taken five minutes after the application. Following this, the participants had free time until their evening visit to the hospital, seven hours later. The participants were instructed not to remove their maxillary dentures during the free-time period.

The participants were allowed to eat and drink whatever they wanted for lunch. However, they were asked to consume similar types and amounts of food and drink during both sessions with adhesives A and B. The third measurement, taken seven hours after the application, was performed when the participants visited our hospital again in the evening.

Data Collection

Measurements of denture movement and subjective evaluation of denture stability were performed at baseline, five minutes after application, and seven hours after application.

Denture movement: Maxillary denture movements during chewing were measured using a commercially available jaw-tracking system (Mandibular Kinesiograph (MKG), K7 evaluation system; Myotronics Research Inc., Kent, WA, USA) [2,16]. The participants sat upright on a dental chair with the Frankfort plane parallel to the horizontal plane. In this study, a small magnet was attached to the labial midline of the maxillary denture. The sensor array was positioned according to the manufacturer’s instructions. Scan number 3 was used with a 1 mm/division setting for vertical, anterior/posterior, and lateral movements, and sweep speeds of 4 sec/division. Vertical movement was used to evaluate denture movements because a previous study reported that the differences in conditions are more easily observed in vertical movement [2]. Once each movement data point was saved in the K7 system, it was captured as an image using the system tool “capture image.” The amplitude of the vertical movement was then measured using image analysis software (ImageJ; National Institutes of Health, or NIH). The first five strokes at the beginning of chewing were discarded because they involved the initial positioning of the test food over the teeth [17]. The peak-to-peak amplitudes of the vertical movements of 15 strokes from the 6th to the 20th strokes were measured. The average value for each measurement condition was then calculated for further statistical analysis.

**Figure 3:**
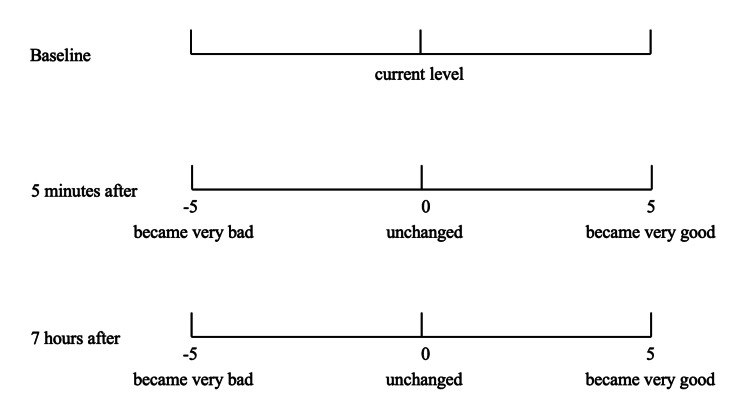
Subjective evaluation for denture stability using a 10 cm VAS (from -5 to 5) VAS, Visual Analog Scale

Subjective evaluation of denture stability: Subjective evaluations were performed using two methods. Subjective evaluations of denture stability were performed at baseline, five minutes after application, and seven hours after application in each session. Baseline values served as references, and the conditions at five minutes and seven hours after application were evaluated using a 10 cm visual analog scale (VAS), ranging from -5 to 5 (Figure 3).

Additionally, a subjective comparison between the two adhesives was performed at the end of the second session using a questionnaire in terms of stability of the denture, duration of stableness, inhibitory effect on wobble, and inhibitory effect on slippage. The participants were asked to choose from five levels of responses. If they preferred the adhesive of the first session over that of the second session, they chose “Prefer the first one than the second one.” If they slightly preferred the adhesive of the first session over that of the second session, they chose “Slightly prefer the first one than the second one.” If they could not decide which was better, they chose “Difference is not found.” This study was carried out as a single-blind and randomized study, therefore, the participants did not inform about which adhesive was used in each session (Table 1).

**Table 1:** Questionnaire about impressions from use (comparison between two adhesives) If the participants preferred the adhesive of the first session over that of the second session, they chose “Prefer the first one than the second one.” If they slightly preferred the adhesive of the first session over that of the second session, they chose “Slightly prefer the first one than the second one.” If they could not decide which was better, they chose “Difference is not found.”

	Prefer the first one than the second one	Slightly prefer the first one than the second one	Difference is not found	Slightly prefer the second one than the first one	Prefer the second one than the first one
Stableness of denture					
Duration of stableness					
Inhibitory effect for wobble					
Inhibitory effect for slippage					

Statistics

Experiment 1

Differences in the rheological characteristics between the two adhesives were compared using a t-test. The normality of the data for temporal changes in adhesion strength was evaluated using the Kolmogorov-Smirnov test. Since some of the 60 cycles demonstrated a non-normal distribution (p < 0.05), the Mann-Whitney U test was used to compare the temporal changes between the two adhesives.

Experiment 2

The normality of the data for denture movements and subjective evaluations of denture stability was assessed using the Kolmogorov-Smirnov test. Since both denture movements (p > 0.11) and subjective evaluation of denture stability (p > 0.09) exhibited a normal distribution, a two-way repeated-measures analysis of variance (ANOVA) was performed, followed by a post hoc comparison using the Tukey test. The factors in the ANOVA were the adhesive type (two levels: adhesives A and B) and time (three levels: baseline, five minutes after, and seven hours after).

IBM SPSS Statistics for Windows, Version 22 (Released 2013; IBM Corp., Armonk, NY, USA), was used for all statistical analyses. Mean values ± SD are provided in the text and figures. Statistical significance was set at p < 0.05.

## Results

Experiment 1

**Figure 4:**
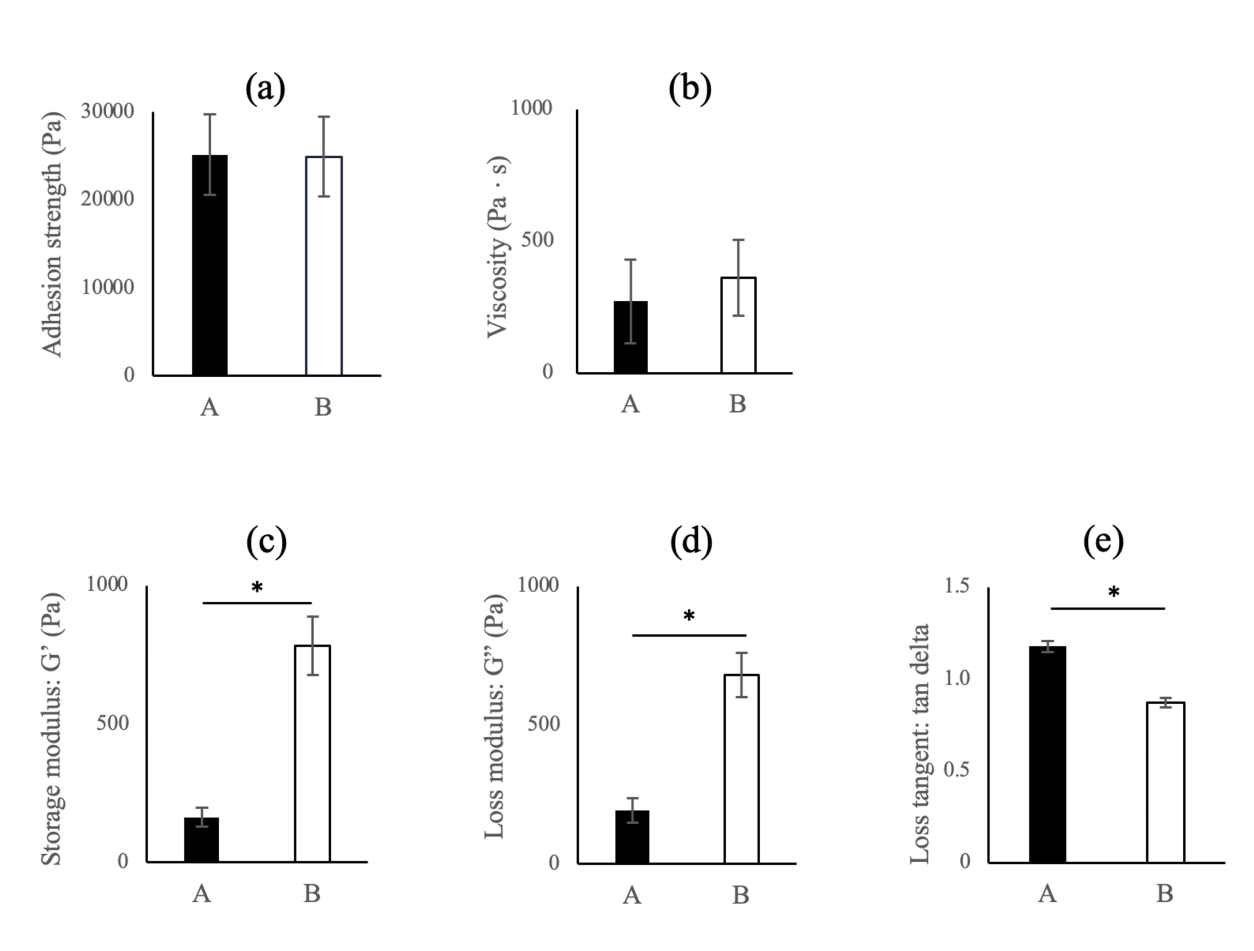
Comparison of material characteristics (a) Adhesion strength; (b) Viscosity; (c) Storage modulus; (d) Loss modulus; (e) Loss tangent Differences between the two adhesives were compared using a t-test. *p < 0.05: significant difference between adhesives A and B.

No significant differences were observed in adhesion strength and viscosity between adhesives A and B (p = 0.94 and p = 0.38 for adhesion strength and viscosity, respectively). The storage modulus, loss modulus, and loss tangent were significantly different between adhesives A and B (p < 0.001 for all three parameters). Adhesive B demonstrated higher storage and loss modulus values than adhesive A. In contrast, the loss tangent was higher for adhesive A than for adhesive B (Figure 4).

**Figure 5:**
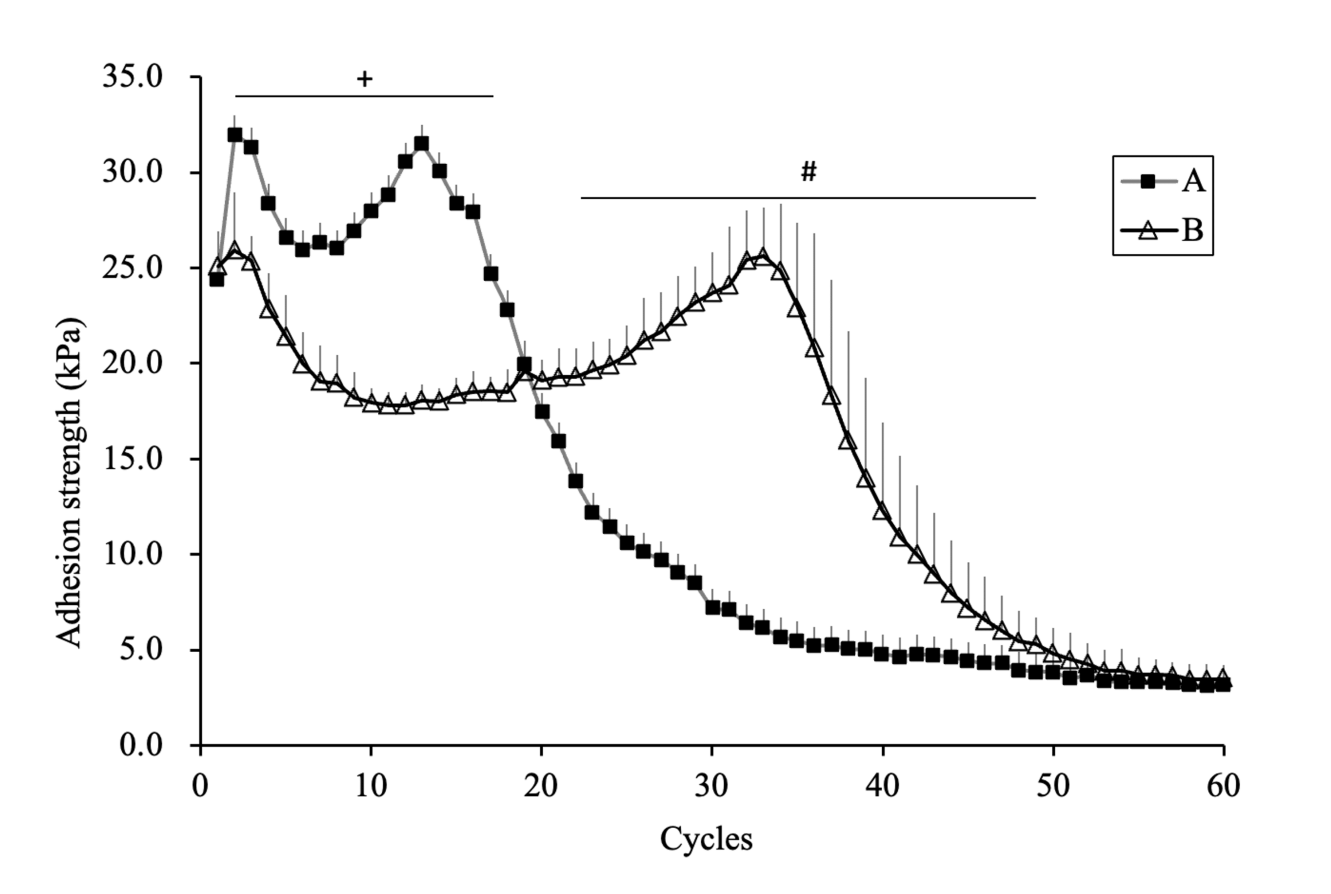
Temporal change of adhesion strength (mean ± SD) "+" denotes A > B (p < 0.05); "#" denotes A < B (p < 0.05). The Mann-Whitney U test was used.

In terms of the temporal changes in the adhesion strength over 60 cycles, from the 2nd to the 17th cycle, adhesive A exhibited significantly higher values than adhesive B (p < 0.05). From the 22nd to the 49th cycle, adhesive B exhibited significantly higher values than adhesive A (p < 0.05) (Figure 5).

Experiment 2

Vertical Denture Movements

**Figure 6:**
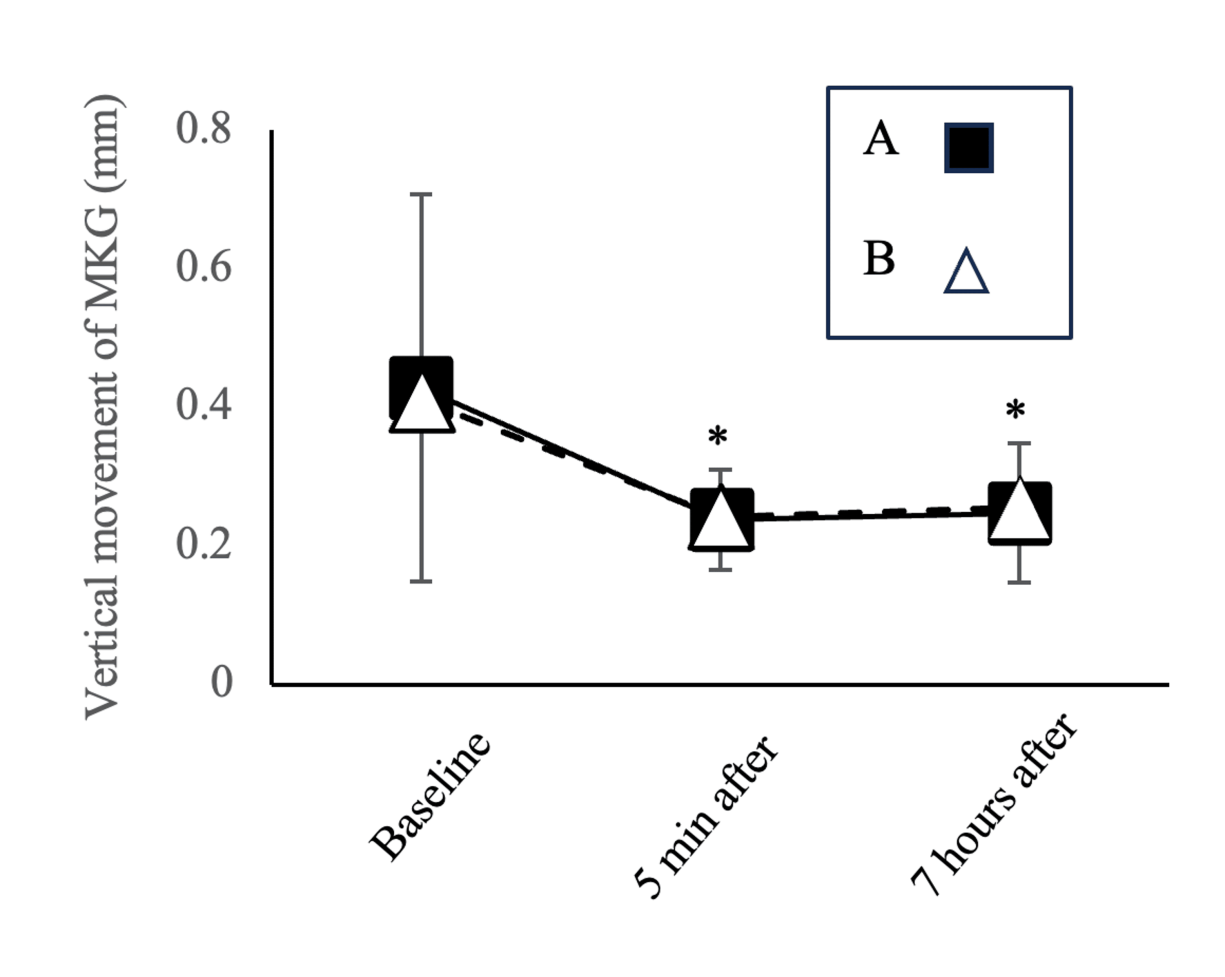
Time course of vertical movement using the MKG system *p < 0.05: significant difference from baseline in the time factor (mean ± SD). A two-way repeated-measures analysis of variance (ANOVA) was performed, followed by a post hoc comparison using the Tukey test. The vertical movement significantly decreased at five minutes (p < 0.01) and seven hours after the adhesive application (p < 0.01) compared to the baseline in both adhesives. No significant differences were observed for the adhesive type (p = 0.96) or the interaction between time and adhesive type (p = 0.84). MKG, Mandibular Kinesiograph

According to the ANOVA results, the time factor was significant (p < 0.01). The vertical movement significantly decreased at five minutes (p < 0.01) and seven hours after the adhesive application (p < 0.01) compared to the baseline in the post hoc test. However, no significant differences were observed for the adhesive type (p = 0.96) or the interaction between time and adhesive type (p = 0.84) (Figure 6).

Subjective Evaluation

**Figure 7:**
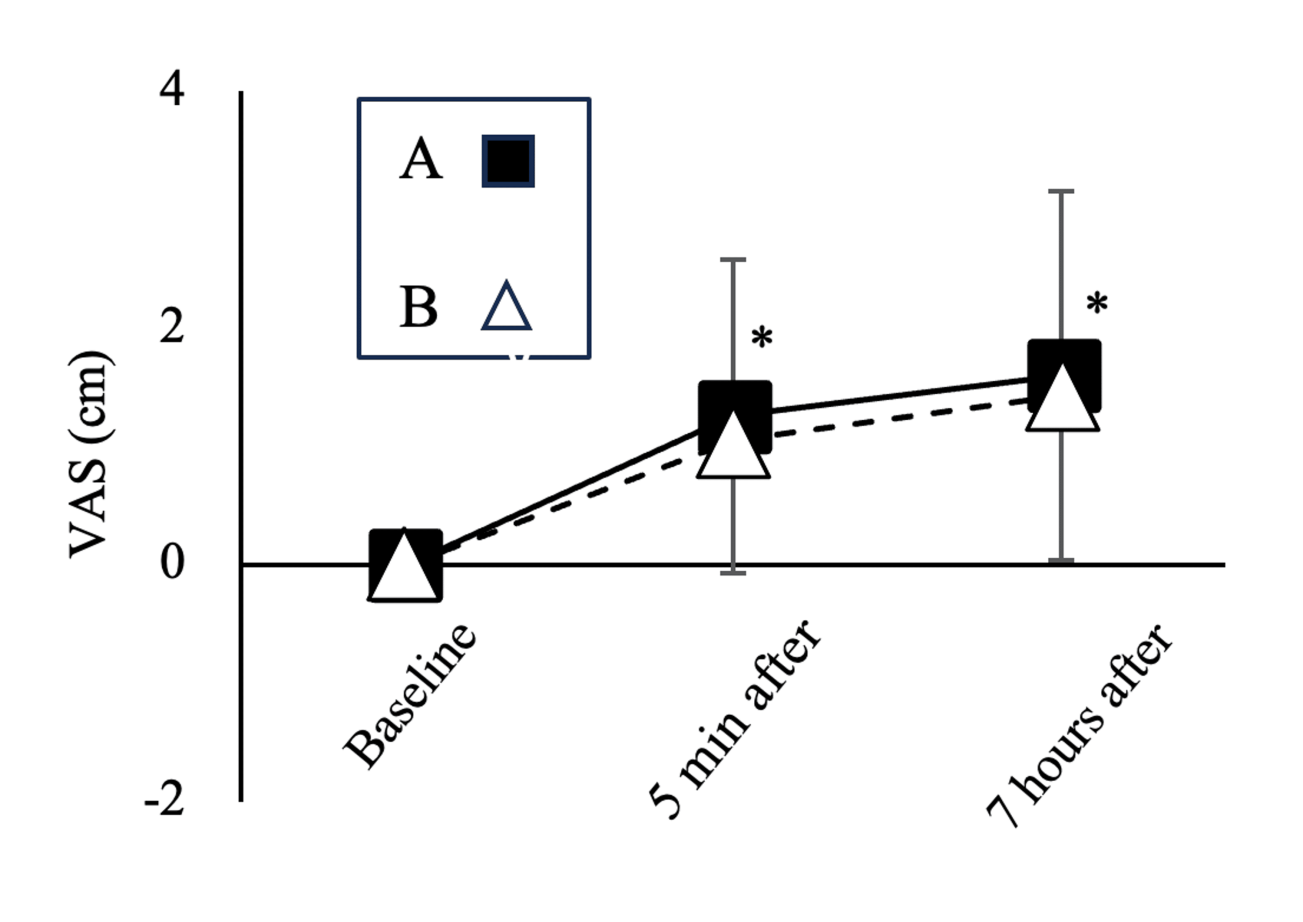
Time course of subjective evaluation (VAS) of denture stability *p < 0.05: significant difference from baseline in the time factor (mean ± SD). A two-way repeated-measures analysis of variance (ANOVA) was performed, followed by a post hoc comparison using the Tukey test. Subjective denture stability significantly increased at five minutes (p < 0.01) and seven hours after the adhesive application (p < 0.01) compared to baseline in both adhesives. No significant differences were observed for the adhesive type (p = 0.70) or the interaction between time and adhesive type (p = 0.91). VAS, Visual Analog Scale

In the ANOVA results, the time factor was significant in the subjective evaluation of denture stability (p < 0.01). Subjective denture stability significantly increased at five minutes (p < 0.01) and seven hours after the adhesive application (p < 0.01) compared to baseline in the post hoc test. However, no significant differences were observed for the adhesive type (p = 0.70) or the interaction between time and adhesive type (p = 0.91) (Figure 7).

Table 2 presents the results of the subjective comparison of the two adhesives. Adhesive B was preferred for its duration of stableness. However, overall, there were no clear differences in the participants' preferences between adhesives A and B.

**Table 2:** Results about impression from use Comparison between the two adhesives using the questionnaire provided (Table 1), with N (%).

	A	Slightly better A	Difference is not found	Slightly better B	B
Stableness of denture	0 (0)	4 (33.3)	3 (25)	4 (33.3)	1 (8.3)
Duration of stableness	0 (0)	2 (16.6)	5 (41.6)	3 (25)	2 (16.6)
Inhibitory effect for wobble	2 (16.6)	2 (16.6)	3 (25)	4 (33.3)	1 (8.3)
Inhibitory effect for slippage	1 (8.3)	4 (33.3)	3 (25)	3 (25)	1 (8.3)

## Discussion

We investigated the differences in material characteristics between two denture adhesives (the existing and improved products) in vitro. Additionally, we investigated denture movement and subjective evaluations after participants used the adhesives for a prolonged period (seven hours). In the in vitro study, no significant differences between the groups were observed in adhesion strength and viscosity at 37°C. However, storage modulus, loss modulus, and loss tangent demonstrated significant differences between adhesives A and B at 37°C. With respect to changes in temporal adhesion strength, adhesive B demonstrated significantly higher values than adhesive A in the later cycles. Therefore, our hypothesis was confirmed only for certain factors.

In the clinical study on long-term use in patients, only the duration of stableness in the questionnaire showed a tendency to favor adhesive B. However, vertical denture movement and subjective evaluations by VAS did not show any statistical differences between the two adhesives. Therefore, our hypothesis was not confirmed in clinical situations, except for the duration of the stableness of subjective feelings.

The vertical movement significantly decreased, and subjective denture stability significantly increased after five minutes compared to the baseline for both adhesives. These effects were maintained seven hours later. Our results indicate that both adhesives have sufficiently long-lasting effects under conditions similar to those in this study.

In terms of material characteristics, there were significant differences in the viscoelastic properties and temporal changes in adhesion strength in vitro between adhesives A and B. However, no differences were observed between the two adhesives in vertical movement in the participants or in the subjective evaluation of denture stability. Similar to our study, some previous studies couldn’t find significant differences between adhesives in vivo studies [6,11,18,19]. Atassi et al. suggested that the findings of in vitro tests for denture adhesive performance are not always translated to in vivo performance [18].

In this study, the gap between the two plates of the stress rheometer was set to 1.0 mm during the measurement of the viscoelastic properties. Moreover, the shaft movement distance was approximately 3 mm during the measurements of the temporal changes in adhesion strength. In a clinical setting, relining of a denture is necessary when the gap between the denture intaglio surface and the denture-bearing mucosa exceeds 0.13 mm [20]. The gaps under the dentures were much smaller than those in the setting of the stress rheometer due to the use of well-fitting dentures in this study. Consequently, the in vitro conditions were more severe than those encountered in daily use, making the adhesives more susceptible to removal. Therefore, no differences between the two adhesives were observed in participants, despite the differences noted in vitro. If ill-fitting dentures had been used or the measurements had extended beyond seven hours, the results might have varied.

However, it is important to note that the gap under ill-fitting dentures in clinical conditions differs from the gap measured by the rheometer. Gaps in the rheometer are necessary for proper operation and vary depending on the type of material used [21]. The material characteristics between two acrylic resin plates were measured in an in vitro test. In contrast, clinical conditions involve one side being acrylic resin and the other being the oral mucosa. Additionally, gaps under dentures change during chewing cycles. These differences may have influenced the outcomes of our study.

It has also been demonstrated that adhesive strength increases exponentially as the thickness of the material layer decreases, and that the effect of material thickness on adhesion strength is much larger than that of the type of material [21]. Well-fitting dentures were used in this study, resulting in thin denture adhesives. The thinness of denture adhesives, therefore, is also likely to contribute to masking the effect of differences in material characteristics on the results of vertical movement and subjective evaluation. Consequently, both adhesives maintained sufficient efficacy even after seven hours in our results.

The effectiveness of denture adhesives is also affected by oral moisture [8], condition of alveolar ridges [7], hardness of test food [5], etc. Further studies are required to clarify the differences between denture adhesives under more severe conditions, e.g., ill-fitting dentures, dry mouth, residual alveolar ridge with severe resorption, or hard test food.

Two factors may also have contributed to the lack of significant differences in vertical movement and subjective evaluation. First, Iida reported that the viscosity of a liquid interposed between two disks plays an important role in their separation. Specifically, higher liquid viscosity requires greater force to separate the disks, as it must overcome both static and dynamic adhesive forces [22]. Therefore, viscosity and adhesive force are likely involved in denture retention and stability. However, the two denture adhesives used in this study did not exhibit significant differences in adhesive strength or viscosity. Second, the viscosity of denture adhesives can affect the degree of spread on the denture-bearing mucosa during application [13]. However, since the two adhesives used in this study did not exhibit any differences in viscosity, it is likely that there was no difference in the spread of the two denture adhesives during the preparation period of five minutes.

Methodological considerations

A previous study reported that natural foods can exhibit differences in consistency and texture [4]. To avoid these variations during the chewing process, artificial materials are typically used. In the present study, gummy jelly - an artificial material - was used as the test food.

Early cycles of mastication are involved in the initial positioning of the test food over the teeth [17]. Marin et al. evaluated the effects of denture adhesives on the movement of maxillary complete dentures using the MKG system. They reported significant changes only in the vertical movement of the MKG [2]. Therefore, we discarded the first five cycles of mastication. Consequently, the 15 vertical movements of the MKG from the 6th to the 20th cycles were used to evaluate denture movement in this study.

The amount used could be further reduced beyond the experimental condition, and a reduction in the amount used may help detect the difference in duration between the two adhesives.

Study limitations

Ohwada et al. studied the effects of denture adhesives on subjective satisfaction and compared them with soft and hard foods [5]. No significant difference was observed between pre- and post-intervention with the denture adhesive when soft food was used as the test food. However, an improvement in perceived chewing ability was observed after using the adhesive when hard food was used. Therefore, they concluded that denture adhesives affect hard foods, which require a large mastication force. However, in the preliminary experiment of the present study, some participants reported pain while chewing hard gummy jelly. Consequently, we chose to use soft gummy jelly as the test food. This choice may have contributed to the finding of no difference between the adhesives in the patient study.

We investigated the effectiveness of adhesives for long-term use; however, the seven-hour duration of this one-day study may not have been sufficient, with only 12 participants. A two-day experiment could have provided a longer assessment period. In the present study, participants were instructed not to remove their dentures until the second visit. However, having participants wear dentures overnight in a two-day study could raise hygiene concerns. Therefore, we adopted a one-day experiment. Future studies with extended durations and more participants may reveal differences between the two adhesives.

We didn't check the validity of the questionnaire used in this study beforehand. This may be another reason why we could not find the differences in the in vivo study. The questionnaire, with appropriate checks about validity, should be used in future studies. 

## Conclusions

In the in vitro study, changes in temporal adhesion strength showed significant differences between the two adhesives. In the clinical study, generally, no significant differences were observed for the adhesive type. Both the existing and improved products demonstrated sufficiently long-lasting effects under these experimental conditions.

The results of this study suggested that denture adhesives contribute to an improved quality of life and self-esteem, even for well-fitting dentures, and also that further studies are required to clarify the differences between denture adhesives under more severe oral conditions.
